# The Search for Cryptic L-Rhamnosyltransferases on the *Sporothrix schenckii* Genome

**DOI:** 10.3390/jof8050529

**Published:** 2022-05-20

**Authors:** Héctor M. Mora-Montes, Karina García-Gutiérrez, Laura C. García-Carnero, Nancy E. Lozoya-Pérez, Jorge H. Ramirez-Prado

**Affiliations:** 1 Departamento de Biología, División de Ciencias Naturales y Exactas, Campus Guanajuato, Universidad de Guanajuato, Noria Alta S/N, Col. Noria Alta, Guanajuato, Guanajuato 360501, Mexico; hmora@ugto.mx (H.M.M.-M.); karina.kg@gmail.com (K.G.-G.); laura_cgc@hotmail.com (L.C.G.-C.); nelppat@hotmail.com (N.E.L.-P.); 2 Unidad de Biotecnología, Centro de Investigación Científica de Yucatán, A.C., Calle 43 No. 130, Col. Chuburná de Hidalgo, Mérida, Yucatan 97205, Mexico

**Keywords:** fungal cell-wall, glycans, rhamnoconjugates, rhamnosyltransferase

## Abstract

The fungal cell wall is an attractive structure to look for new antifungal drug targets and for understanding the host-fungus interaction. *Sporothrix schenckii* is one of the main causative agents of both human and animal sporotrichosis and currently is the species most studied of the *Sporothrix* genus. The cell wall of this organism has been previously analyzed, and rhamnoconjugates are signature molecules found on the surface of both mycelia and yeast-like cells. Similar to other reactions where sugars are covalently linked to other sugars, lipids, or proteins, the rhamnosylation process in this organism is expected to involve glycosyltransferases with the ability to transfer rhamnose from a sugar donor to the acceptor molecule, i.e., rhamnosyltransferases. However, no obvious rhamnosyltransferase has thus far been identified within the *S. schenckii* proteome or genome. Here, using a Hidden Markov Model profile strategy, we found within the *S. schenckii* genome five putative genes encoding for rhamnosyltransferases. Expression analyses indicated that only two of them, named *RHT1* and *RHT2*, were significantly expressed in yeast-like cells and during interaction with the host. These two genes were heterologously expressed in *Escherichia coli*, and the purified recombinant proteins showed rhamnosyltransferase activity, dependent on the presence of UDP-rhamnose as a sugar donor. To the best of our knowledge, this is the first report about rhamnosyltransferases in *S. schenckii*.

## 1. Introduction

Currently, fungal infections are a worldwide burden to humanity, directly and indirectly. Directly, fungi cause over 1.6 million human deaths annually, similar to the tuberculosis death toll, and more than a billion infections [1,2]. Indirectly, although most fungal colonizations of plants are advantageous symbiotic relationships, devastating fungal diseases account for harvest losses of around 30% of crops of rice, wheat, maize, potatoes, and soybean, five of the top sources of food globally [2]. No less important are the effects caused to ecosystems by fungal infections, destroying entire populations of bats, amphibians, reptiles, bees, and even corals, to cite a few [2]. There is a limited set of antifungal drugs, and some of the medically approved ones are also used in crop fields, a practice that could result in the emergence of drug-resistant strains of human pathogens [3,4], such as the multidrug-resistant pathogen *Candida auris* [5]. Thus, there is an urgent need to find new molecular targets on fungi for better disease control.

The fungal infectious disease process requires a complex set of metabolic pathways along with its regulatory components. Numerous studies have elucidated many of the proteins directly involved in this process, giving high priority to effector molecules, named virulence factors, but neglecting the relevance of other molecules such as cell wall glycans [6,7,8,9]. The main cell wall constituents are carbohydrate-containing molecules (polysaccharides and glycoproteins), pigments, inorganic salts, and lipids, linked by covalent and non-covalent bonds [10,11,12,13,14,15]. Some glycoproteins present on the fungal cell wall have been demonstrated to be required for pathogenicity and are absent from mammalian cells [16,17,18,19]; therefore, the genes required for their biosynthesis represent potential molecular targets to develop new medically relevant antifungal drugs. Even though other wall components are not by themselves virulence factors, glycoproteins are considered virulence determinants because the disruption of their biosynthesis affects cell fitness and, thus, the host-fungus interaction; this is the case of *N*-linked and *O*-linked glycans attached to cell wall proteins [9,13,20,21,22,23]. For example, rhamnose-based glycans are a minor cell wall component and therefore were not previously thought to contribute to an infecting process, but it was found that their synthesis is required for the pathogenicity of the vascular wilt fungus *Verticillium dahliae* [24]. Among the medically relevant fungal species, rhamnose has been currently reported in the genera *Scedosporium/Pseudallescheria* and *Sporothrix.* In *Scedosporium/Pseudallescheria boydii*, this sugar has been reported to be part of peptidorhamnomannan, a cell wall component involved in conidia germination, viability, uptake by macrophages, and survival in a murine model of infection [25]. Similarly, our group has recently reported that rhamnose synthesis is required for normal cell wall composition and *Sporothrix schenckii*-host interaction [26]. *S. schenckii RmlD* silencing disrupted the UPD-L-rhamnose synthesis; and as a consequence, the fungal cell wall *N*-linked and *O*-linked glycans were depleted of rhamnose, and the levels of the latter were significantly reduced [24]. In addition, the β-1,3-glucan levels and exposure at the cell wall surface were positively affected upon disruption of protein rhamnosylation [24]. These cell wall changes led to abnormal proinflammatory and anti-inflammatory cytokine stimulation by human peripheral blood mononuclear cells, increased uptake by human monocyte-derived macrophages, and reduced ability to kill the alternative host *Galleria mellonella* [24]. Even though the role of rhamnose-containing glycoconjugates has not been addressed in other *Sporothrix* species, this monosaccharide is found in the cell wall of *Sporothrix brasiliensis* and *Sporothrix globosa* [10,14,15,27], and a correlation between the glucan-rhamnose ration and virulence has been proposed at least for *S. schenckii* and *S. brasiliensis* [27]. This organism is the etiological agent of human and animal sporotrichosis and belongs to a handful of medically relevant fungal species that contain rhamnose at the cell wall [10,14,18,26,28]. The fungal biosynthetic pathway of rhamnose-containing glycans is currently unknown, but it is assumed that it should include enzymes with the ability to transfer the sugar to a donor molecule using a glycosyltransferase [28,29,30].

Glycosyltransferases (GTs; EC 2.4.x.y) have been found extensively on prokaryotes and eukaryotes and encompass a very diverse family of enzymatic functions required for the biosynthesis of glycoconjugates, polysaccharides, and oligosaccharides [31]. GTs that catalyze the transfer of a sugar residue from an activated nucleotide sugar donor to a specific acceptor molecule are the most abundant but are not the only ones; some enzymes may use lipid diphosphate-sugars, sugar-1-phosphates, and dolichol-phospho-sugars as activated donors [32,33].

In the *Sporothrix schenckii* genome, genes involved in the biosynthesis of UDP-L-rhamnose, the donor of the required monosaccharide for the synthesis of rhamnoconjugates, have been identified [26,30], but despite the availability of *S. schenckii* genomic sequence [30] and transcriptome [34], the L-rhamnosyltransferase gene required for rhamnoconjugation has not been identified through conventional homology searches (BLAST) nor with available Hidden Markov Models profiles (hmm). Here, to search for the elusive genes encoding for rhamnosyltransferases, we designed a robust bioinformatic strategy capable of selectively identifying these enzymes on the *S. schenckii* genome, and the candidate genes were biochemically tested to prove the predicted enzymatic function.

## 2. Materials and Methods

### 2.1. Hardware and Software Environment Used

All locally run bioinformatics analyses were conducted on the Ubuntu 20.04.4 LTS operating system. Where required, analyses were run on publicly accessible servers: NCBI databases [35], PFAM database [36], RCSB PDB database [37,38], and AlphaFold2 [39] mounted on Google Colab projects: AlphaFold2.ipynb and AlphaFold.ipynb [39,40,41,42,43].

### 2.2. Sporothrix Schenckii Proteome and PFAM/TIGRFAM Hidden Markov Models Profiles

The 10,293 proteins of the sequenced *S. schenckii* (ATCC MYA-4821) genome [30] were downloaded from NCBI (Bioproject PRJNA218070).

Available HMM profiles for rhamnosyltransferases, as well as general glycosyltransferases profiles, were downloaded from PFAM and TIGRFAM.

### 2.3. Construction of Ad Hoc Eukaryotic Rhamnosyltransferases HMM Profile and Hmmer Searches

Selected protein sequences of reported eukaryotic rhamnosyltransferases from viridiplantae were downloaded from the NCBI database, and a Multiple Sequence Alignment (MSA) was constructed with the MAFFT algorithm (v.7.450) [44].

Accession numbers of used protein sequences are as follows: NP_564357.1, ACX70154.1, BAC43110.1, AEE31240.1, AAL06646.2, AFB73772.1, Q8GVE3.2, XP_004485549.1, XP_004485550.1, NP_001274799.1, Q9S9P6.1, CAB78073.1, Q66PF2.1, ABB84472.1, BAA98174.1, and XP_014517591.1.

An ad hoc HMM profile was generated from the MSA of the sixteen eukaryotic rhamnosyltransferases with HMMER (v.3.3).

Searches with PFAM/TIGRFAM HMM profiles as well as the ad hoc HMM profile were conducted on the *S. schenckii* proteome using HMMER (v.3.3). The *S. schenckii* proteome was used as the target database.

To determine putative domains present on the recovered sequences, these sequences were submitted to the sequence search tool at the PFAM database, with an E-value cut-off of 1.0.

### 2.4. In Silico Structural Analysis of Putative Rhamnosyltransferases Proteins

Retrieval of the closest crystalized proteins of Rht1 and Rht2 from the RCSB PDB database was performed using its advanced sequence search tool. Default parameters were used except for the “Return” value, which was changed to “Polymer Entities”.

Three-dimensional modeling of Rht1 and Rht2 proteins was carried out at publicly available implementations of the Alphafold2 algorithm [39]. All default parameters were used, and no templates were selected.

Pymol (v.3.2) was used to visualize the 3D protein models. Secondary structures were colored using the default color-scheme (alpha helixes red, beta sheets yellow, coils green). Confidence values for each atom were shown using the default color-scheme (traffic-light-like red-yellow-green gradient).

Alignments of 3D models and crystalized proteins (atoms superimposition) were conducted also on Pymol.

Matching scores and root mean square deviation (RMSD) values were recorded for each pair of structures.

A more detailed version of all bioinformatic methodologies and tools used can be found in Supplementary Materials Bioinformatics Methodologies: Bioinfo_Methods.docx.

### 2.5. Strains and Culture Media

The *S. schenckii* strain ATCC MYA-4821, whose genome has been already sequenced [30], was used in this study. Hyphae were propagated at 28 °C in YPD, pH 4.5 medium (1% [*w*/*v*] yeast extract, 2% [*w*/*v*] gelatin peptone, and 3% [*w*/*v*] dextrose); whereas yeast-like cells were obtained in YPD, pH 7.8, as described elsewhere [14]. Fungal growth in brain-heart infusion (BHI), potato dextrose broth (PDB), Vogel media, and *Galleria mellonella* larva was carried out at 37 °C, as previously described [45]. For heterologous gene expression, *Escherichia coli* BL21 (DE3) from Invitrogen was used. Bacteria were grown in LB broth (0.5 [*w*/*v*] yeast extract, 1% [*w*/*v*] gelatin peptone, and 0.5% [*w*/*v*] NaCl) at 37 °C. When required, broths were supplemented with 2% (*w*/*v*) agar for solid plates.

### 2.6. Analysis of Gene Expression

Total RNA was isolated as reported [46], and total cDNA was synthesized and purified by adsorption chromatography with an in-house methodology previously reported [47]. The cDNA was quantified in a NanoDrop 2000 (Thermo Fisher Scientific, Waltham, MA, USA) and amplified with the SYBR Green PCR Master Mix (Life Technologies, Carlsbad, CA, USA) in a thermocycler StepOne Plus (Life Technologies). Relative gene expression was calculated with the StepOne software V 2.2 (Life Technologies) using the 2^−^^∆∆Ct^ method [48]. The encoding gene for the ribosomal protein L6 was used as an endogenous control [45], while the filament growth in YPD at 28 °C was defined as the reference condition. The following primer pairs were used in the RT-qPCR reactions: 5′-ATTGCGACATCAGAGAAGG-3′ and 5′-TCGACCTTCTTGATGTTGG-3′ for the endogenous control; 5′- AACAGATTGAGGCTCTGGGC-3′ and 5′-AGTAAGTCTGGGTTCGCCAC-3′ for SPSK_05538; 5′-CAGAACGGAAAGACGGGACA-3′ and 5′-ACCACCGTTGTTGTTGACCT-3′ for SPSK_05928; 5′-AGGAATGACCATCAGCGCAA-3′ and 5′-AAACGCTCGGGAAAGTCCAT-3′ for SPSK_01368; 5′-AGCCACGAACGTCTCTTCAG-3′ and 5′-CTTCAAACGTGCCTGATGGC-3′ for SPSK_04821; and 5′-GTTTGTCCCCGACGTTTTGG-3′ and 5′-GCCGCGAGATGAAAAAGACG-3′ for SPSK_01110.

### 2.7. Heterologous Gene Expression in Escherichia coli and Protein Purification

The open reading frames under analysis were amplified by PCR using the primer pairs 5′-GAATTCATGGGCCCGGGCAAGAACC-3′ and 5′-TCTAGACTAGTCTAGTCGCAGCAGA-3′ for SPSK_05538; and 5′-GAATTCATGAAGGTCCTCGTGTCCAC and 5′-TCTAGA TTACGACTGAGCACTCTCGGC for SPSK_01110 (underlined bases indicate EcoRI and XbaI sites, added to the primer sequences, to direct cloning). The 699 bp and 1731 bp amplicons were cloned into pCR2.1-TOPO (Invitrogen, Waltham, MA, USA) and then subcloned into the EcoRI and XbaI sites of pCold I (Takara Bio Inc., San Jose, CA, USA), generating pCold-RHT1 and pCold-RHT2, respectively. For the expression of recombinant genes, cells were grown in LB broth with 100 µg mL^−1^ of ampicillin (Sigma-Aldrich, San Luis, MO, USA) for 20 h at 37 °C and orbital shaking (200 rpm). Then, 150 mL of fresh broth contained in a 2-L Erlenmeyer flask was inoculated with 1.5 mL of the overnight culture and incubated at 37 °C until reaching an O.D.600 nm = 0.4; then, 0.1 mM of isopropyl-β-D-1-thiogalactopyranoside was added, and it was further incubated for 20 h at 20 °C. Cells were harvested by centrifuging for 20 min at 1500× *g* and 4 °C kept at −20 °C until use.

For protein purification, induced bacteria were washed three times with PBS, resuspended in 5 mL of the same buffer added with 0.5 mg mL^−1^ lysozyme (Sigma-Aldrich), and incubated for 30 min at 37 °C. Then, cells were resuspended in 10 mM Tris-HCl, pH 7.4, 1 mM EDTA, and 1% (*w*/*v*) SDS and disrupted by incubating for 30 min at 37 °C and shaking for 20 min in a vortex. The cell homogenate was dialyzed in a cellulose membrane (Sigma-Aldrich) for 24 h, concentrated in an Amicon Ultra centrifugal filter with Ultracel-3K (Sigma-Aldrich), and purified with the Talon Metal Affinity Resin (Takara Bio Inc.), as suggested by the manufacturer. Then, the protein was dialyzed and concentrated in an Amicon Ultra-4 system [49]. SDS-PAGE was used to analyze the purity of samples. Aliquots of 30 µL were placed onto 12% (*w*/*v*) gels, and protein separation was performed at 120 V for 90 min. The densitometric analysis in a ChemiDoc™MP (Bio-Rad, Hercules, CA, USA) system of silver-stained protein bands [50] was used to assess the protein purity of the recombinant proteins, while the Pierce BCA Protein Assay (Thermo Fisher Scientific) was used for protein quantification.

### 2.8. Rhamnosyltransferase Assays

Enzyme activity was assayed as previously described [51], using 200 ng α-1,6-mannobiose (Dextra Laboratories, Reading, UK) as the rhamnose acceptor and 500 µM UDP-L-rhamnose (Chemily Glycoscience; Peachtree Corners, GA, USA). The reaction products were analyzed by High-Performance Anion-Exchange Chromatography with Pulsed Amperometric Detection (HPAEC-PAD) with a Dionex system (Thermo Fisher Scientific), using a CarboPac PA-100 column (4.6 × 250 mm) and a linear gradient of 10–100 mM sodium acetate in 100 mM NaOH at a flow rate of 0.8 mL min^−1^ for 30 min. [52]. For assays including treatment with α-L-rhamnosidase, the reaction product of rhamnosyltransferase activity was mixed with 1 U enzyme (Megazyme, Bre, Ireland) and incubated for 60 min at 50 °C. The reactions were stopped by boiling for 10 min and subjected to monosaccharide separation by HPAEC-PAD using the following conditions: a CarboPac PA-200 analytical column (3 × 250 mm) with a CarboPac PA-200 guard column (3 × 50 mm) and an isocratic gradient of 3.2 mM NaOH with a flux rate of 0.15 mL min^−1^ for 25 min [53]. Fluorescence-assisted carbohydrate analysis (FACE) was performed essentially as previously described [54]. UDP-glucose, GDP-mannose, UDP-N-acetylglucosamine, and UDP-galactose were from Sigma-Aldrich.

### 2.9. Statistical Analysis 

Statistical analysis was performed using GraphPad Prism 6 software. Enzyme activity assays were performed at least three times in duplicate and were analyzed with the unpaired *t*-test to establish statistical significance. All data are represented as the mean and standard deviation. In all cases, the significance level was set at *p* < 0.05.

## 3. Results

### 3.1. Identification of Putative Genes Encoding for Rahmosyltranferases

When applying the rhamnosyltransferase specific PFAM and TIGRFAM profiles (PF11316 and TIGR01556) to the *S. schenckii* proteome, no significant results were retrieved. To overcome the limitations of using the prokaryotic HMM profile on a eukaryotic genome, we created a specific profile to search for putative eukaryotic rhamnosytransferases. For this purpose, we selected reported rhamnosyltransferases from plants (*Arabidopsis thaliana*, *Cicer arietinum*, *Citrus maxima*, *Solanum tuberosum*, and *Vigna radiata*) and built an HMM profile from their multiple sequence alignment (Supplementary Materials file Rhamno_Transf_Euk.hmm). We then proceeded to search the *S. schenckii* proteome using this HMM profile, recovering five protein sequences (SPSK_01368, SPSK_04821, SPSK_05538, SPSK_05928, and SPSK_01110) ranging in sizes from 232 aa to 1836 aa (Table 1). Four of the five sequences were automatically annotated as glycosyltransferases, and a fifth sequence was only annotated as a hypothetical protein (Table 1).

The five sequences were then scanned against the whole Pfam HMM profile database [36] to identify putative domains/motifs present. As shown in Table 2, three HMM profiles related to glycosyltransferases were identified: PF00201 (UDP-glucoronosyl and UDP-glucosyl transferase), PF04101 (Glycosyltransferase family 28 C-terminal domain), and PF03033 (Glycosyltransferase family 28 N-terminal domain). The predicted positions of these putative domains on the protein sequences are shown in Table 2, as well as the confidence levels expressed as individual and conditional E-values. Although none of the HMM profiles were predicted for all five sequences, all of them are part of the Pfam CL0113 (GT-B) clan of glycosyltransferases that possess two Rossmann-like folds and lack the DxD motif, contrary to typical prokaryotic GT-A rhamnosyltransferases.

Therefore, these five putative genes were selected for further analysis.

### 3.2. Expression Analysis of Five Putative Rhamnosyltransferases on Sporothrix Schenckii

**Figure 1:**
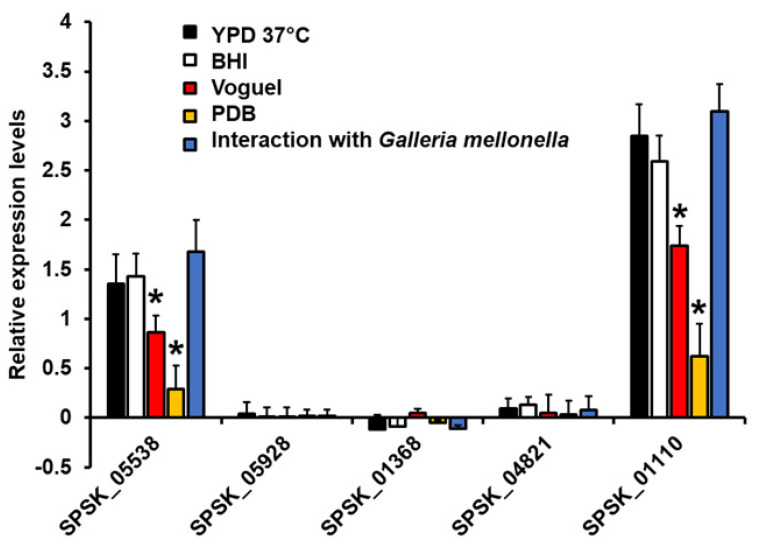
Analysis of gene expression. Cells were grown in YPD, BHI, Voguel, or PDB medium at 37 °C, and total RNA was extracted; cDNA was synthesized with oligo(dT) primer (20 mer) and quantified by RT-qPCR. Alternatively, *Galleria mellonella* larvae were inoculated with 1 × 10^6^ yeast-like cells, and, after 72 h of interaction at 37 °C, fungal cells were retrieved and used for total RNA extraction. Expression levels were normalized using the gene encoding for the ribosomal protein L6 as the control and the fungal growth in YPD at 28 °C as reference conditions. Results are means ± SD of three independent experiments performed by duplicate. * *p* < 0.05 when compared to the expression levels of the same gene in the other growing conditions.

To assess whether these sequences are indeed true genes, we first analyzed expression in different growth conditions. The gene encoding for ribosomal protein L6 and the filament growth in YPD broth were used as the reference gene and condition, respectively, for gene expression analysis [45]. The SPSK_05538 and SPSK_01110 sequences were found to be expressed in yeast-like cells growing in YPD broth, indicating that these are indeed genes (Figure 1). The SPSK_01110 gene was found to be overexpressed in these growing conditions when compared to the SPSK_05538 gene, and a similar trend was observed in yeast-like cells growing in BHI or collected from infected *G. mellonella* larvae at day three post inoculation (Figure 1). Interestingly, the expression levels of both genes were significantly reduced when cells were grown in Vogel medium, which favors the growth of both mycelia and swollen yeast-like cells [45], and this expression was even lower in cells grown in PDB, a medium that precludes the morphological transition from filament cells to yeast-like cells [45] (Figure 1). The sequences SPSK_05928, SPSK_01368, and SPSK_04821 showed minimal expression levels in all the tested conditions (Figure 1), suggesting that their expression was not strong and influenced by the dimorphism. It has been previously reported that cell wall rhamnose levels are influenced by the dimorphism, being higher in yeast-like cells than in germlings [14]; therefore, in this work, we decided to continue working with the only two genes showing gene regulations by dimorphism and growth conditions, SPSK_05538 and SPSK_01110. These genes were renamed Rhamnosyltransferase 1 (*RHT1*) and Rhamnosyltransferase 2 (*RHT2*), respectively.

### 3.3. Sporothrix schenckii RHT1 and RHT2 Expression in Escherichia coli and Characterization of the Enzyme Activity

**Figure 2:**
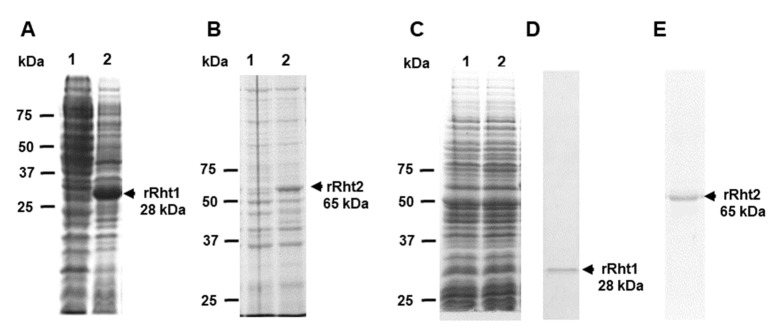
Electrophoretic profile of recombinant Rht1 and Rht2 expressed in *Escherichia coli*. Bacteria were transformed with pCold-RHT1 (**A**), pCold-RHT2 (**B**), or empty pCold I (**C**) and grown under non-inducive conditions (lanes 1) or in inducing conditions (lanes 2, 0.1 M isopropyl-β-D-1-thiogalactopyranoside and 20 h at 20 °C). Protein samples were prepared from cultured cells and separated by denaturing SDS-PAGE. The recombinant proteins rRht1 (**D**) and rRht2 (**E**) were then subjected to purification by affinity chromatography using a cobalt-charged resin. Panel **A** corresponds to an electrophoretic analysis in 12% acrylamide gel, while gels of 10% acrylamide were used in panels (**B**–**E**).

Next, the coding sequences of *RHT1* and *RHT2* were cloned into the expression vector pCold I, generating pCold-RHT1 and pCold-RHT2, respectively. These constructions were used to transform *E. coli* cells, and gene expression was induced with isopropyl-β-D-1-thiogalactopyranoside, with incubation at 20 °C. Several conditions for gene induction were tested in pilot experiments (not shown), and the best conditions were found at 0.1 M of the inductor and 20 h of incubation. Under these inducing conditions, a 28 kDa recombinant protein was induced in cells transformed with pCold-RHT1, a molecular weight closer to the 25 kDa predicted molecular weight for the *RHT1* encoded peptide (Figure 2A). For the case of cells transformed with pCold-RHT2, a protein band of 65 kDa was generated when grown under inducing conditions (Figure 2B), and this molecular weight was similar to 62 kDa, the predicted molecular weight for the product of *RHT2*. Since the expression vector contained sequences for molecular tags that are part of the recombinant proteins, such as 6xHis [49], it is likely that these extra sequences were responsible for the discrepancy between the experimental and predicted molecular weights. Cells growing in non-inducive conditions or transformed with the empty pCold I vector did not show the differentially expressed protein bands (Figure 2A–C), suggesting that those protein bands of 28 and 65 kDa were the recombinant (r)Rht1 and rRht2, respectively. Since both proteins contained the 6xHis tag at the N-terminal end, protein purification was performed by affinity chromatography in a cobalt-charged resin. Following this strategy, one single protein band was observed in the purified fractions, with the corresponding 28 and 65 kDa for rRHT1 and rRHT2, respectively (Figure 2D,E). The densitometric analysis of the silver-stained gel subjected to SDS-PAGE confirmed that more than 98.5% of the protein present in the preparations corresponded to the recombinant proteins. Following this procedure, we obtained a yield of 16.1 ± 3.2 µg and 19.4 ± 2.7 µg pure rRht1 and rRht2 per mL of induced culture media, respectively.

**Figure 3:**
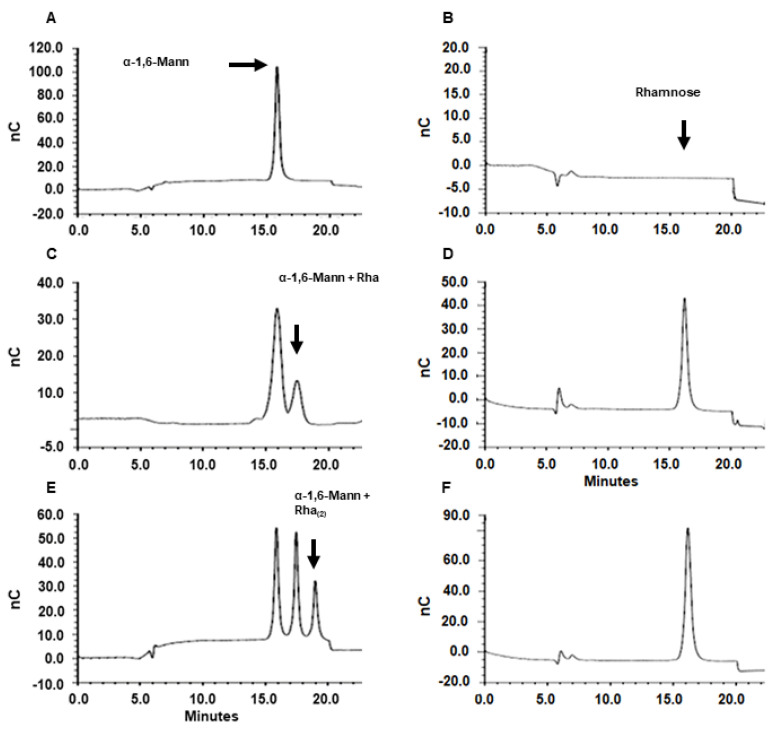
Enzyme activity of recombinant Rht1. Reaction mixtures containing the purified enzyme (100 µg protein), 200 ng α-1,6-mannobiose, and 500 µM UDP-L-rhamnose were incubated at 37 °C. After the indicated times, samples were heated in boiling water and analyzed by HPAEC as described in the text. Panels (**A**,**C**,**E**) correspond to the incubation times of 0 min, 30 min, and 3 h, respectively. The enzyme product was treated with 1 U α-L-rhamnosidase for 60 min, and the monosaccharides released were analyzed by HPAEC. Panels (**B**,**D**,**F**) correspond to monosaccharides released from enzyme products obtained at the incubation times of 0 min, 30 min, and 3 h, respectively.

Next, to demonstrate the rhamnosyltransferase activity of the purified enzymes, we set up a detection system based on α-1,6-mannobiose as the sugar acceptor and UDP-L-rhamnose as a donor. We selected these molecules because this disaccharide has been found as a part of the natural rhamnose-containing *N*-linked glycans found in *S. schenckii* peptidorhamnomannan, and UDP-conjugated but not dTDP-rhamnose has been recently identified as the rhamnose donor in this organism [18,26]. The product reactions were then analyzed by HPAEC-PAD. Figure 3 shows the analysis of the rRht1 products. At time 0 of the incubation at 37 °C, only one major peak at 16.1 min was detected, and this corresponded to the acceptor α-1,6-mannobiose (Figure 3A). After 30 min incubation, a second peak with a retention time of 17.8 min was also observed, and its quantity increased when reactions were incubated for 3 h (Figure 3B,C). This result suggested that it was a product of the rhamnosyltransferase activity. To confirm that this is indeed rhamnosylated mannobiose, this enzyme product was subjected to derhamnosylation by treatment with α-L-rhamnosidase, and the released sugars were again analyzed by HPAEC-PAD to detect released monosaccharides. At time 0, no monosaccharide was detected in the preparations, indicating that all the rhamnose was bound to UDP (Figure 3B). However, after 30 min incubation and subsequent treatment with rhamnosidase, rhamnose was detected in the samples, suggesting that this monosaccharide was released from mannobiose (Figure 3D), and the amount of the detected monosaccharide increased in preparations incubated for 3 h with rhamnosyltransferase (Figure 3E). In agreement, the oligosaccharide analysis of the derhamnosylated samples only showed the peak corresponding to mannobiose (data not shown). The α-L-rhamnosidase was incapable of releasing the monosaccharide from UDP (data not shown), underlining that the detected rhamnose was bound to another acceptor, i.e., mannobiose. Control reactions with denatured rhamnosyltransferase by boiling or bacteria homogenates subjected to the same purification procedure failed to generate the second peak observed in panels C and D of Figure 3. Moreover, FACE analysis showed that this enzyme product had mobility similar to a trisaccharide (data not shown). It was interesting to observe that, after 3 h of incubation, an extra peak with a retention time of 19.2 min was generated in the reactions (Figure 3D). Once again, upon derhamnosylation, this peak disappeared from the preparations, and it was shown that only mannobiose and free rhamnose was detected, and the FACE analysis was suggested to be a tetrasaccharide (data not shown and Figure 3 E). Similar results were obtained with rRht2 (not shown). Collectively, this strategy showed that both enzymes were capable of transferring rhamnose from UDP to the acceptor α-1,6-mannobiose.

The analysis under the curve allowed us to generate quantitative data from these assays, and we found that rRht1 and rRht2 showed a specific activity of 198.7 ± 34.4 and 215.4 ± 28.6 µg trisaccharide min^−1^ mg protein^−1^ (Table 3). Mock reactions with no sugar donor or with bacterial extract subjected to the same purification procedure gave threshold values (Table 3). In addition, the enzyme activity was minimal when the heat-denatured enzyme was included in the reactions (Table 3). Next, to assess the specificity of the enzyme for the sugar donor, UDP-rhamnose was substituted by equimolar concentrations of UDP-glucose, GDP-mannose, UDP-N-acetylglucosamine, and UDP-galactose. None of these molecules were recognized by rRht1 or rRht2 as sugar donors in the enzyme reaction (Table 3), suggesting that both enzymes are specific for UDP-rhamnose.

### 3.4. In Silico Structural Analysis of Sporothrix Schenckii Rht1 and Rht2 Proteins

**Figure 4:**
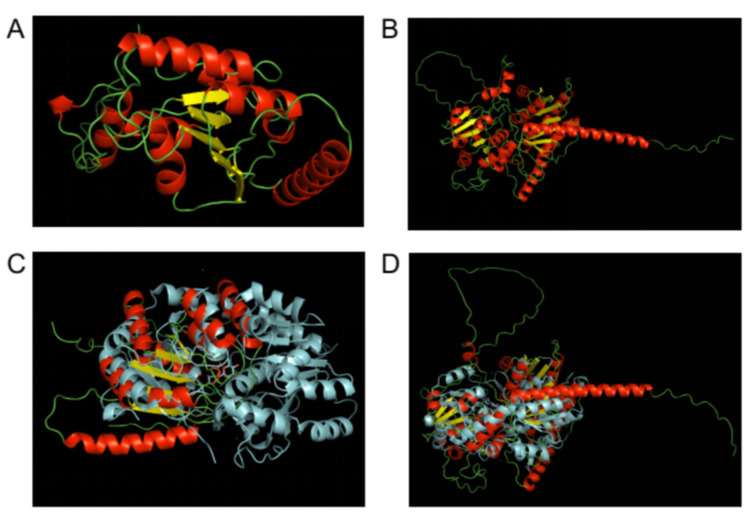
In silico structural analysis of *Sporothrix schenckii* Rht1 and Rht2 proteins. Alphafold2 predicted models for Rht1 (**A**) and Rht2 (**B**) and rendered on PyMol. Secondary structures are color-coded as follows: alpha helixes red, beta sheets yellow, coils green. Alignments (atoms superimposed) of the predicted 3D model to *Arabidopsis thaliana* rhamnosyltransferase UGT89C1: Rht1/UGT89C1 panel (**C**) and Rht2/UGT89C1 panel (**D**). Rht1 (**C**) and Rht2 (**D**) are colored according to secondary structure, while UGT89C1 is shown in cyan.

To further characterize the two identified and proven rhamnosyltransferase genes, we conducted a structural analysis of the two predicted proteins. De novo tridimensional folding of the proteins was conducted through the Alphafold2 algorithm [39], and the best predicted models were recovered for each protein sequence. The predicted 3D models of both proteins Rht1 and Rht2 (Figure 4A,B, respectively) showed the characteristic α/β/α Rossmann nucleotide binding domains [55,56], but Rht1 appears to have a single one, while Rht2 has the two Rossmann-fold characteristics of GT-B glycosyltransferases. Confidence levels per residue for predicted Rht1 and Rht2 protein 3D structures, expressed as predicted LDDT (pLDDT), are visually represented as color-coded renderings of the protein predictions on panels A and B, respectively, and graphed per position on panels C and D, respectively, of the Supplementary Materials Figure S1. For both predictions, most of the secondary structure conforming the α/β/α Rossmann domains show pLDDT confidence values > 90, which were expected to be modeled to high accuracy. Although some short regions had pLDDT values between 70 and 90, these were still expected to be modeled well, and represented a generally good backbone prediction. Regions for Rht2 with low pLDDT values (<30) corresponded to the flexible linker between the two Rossmann domains. Additionally, the predicted model for Rht1 had low confidence values at both the N-terminal and C-terminal ends (Supplementary Materials Figure S1C,D).

As a mean to corroborate the correctness of the modeled 3D structures, we aimed to compare those models to reported crystalized structures. For this, we searched the Protein Data Bank (RCSB PDB) for crystal structures of known rhamnosyltransferases, as well as for crystal structures of proteins that shared high sequence similarity to use as templates to compare those structures to the predicted 3D models of Rht1 and Rht2. At amino acid level, Rht1 shares its closest similarity to CalG1 (PDB ID 3OTG; [57]), a GT-B fold Calicheamicin Glycostyltransferase (Sequence Identity: 21%, E-Value: 0.000002912, Region: 21–411) from the Actinomycetota *Micromonospora echinospora*. Correspondingly, the sequence of Rht2 is most similar to that of Alg13 (PDB ID 2JZC; [58]) the sugar donor subunit of *Saccharomyces cerevisiae* GT-B fold N-acetylglucosamine transferase (Sequence Identity: 32%, E-Value: 5.177e-12, Region: 32–180). When the 3D models of Rht1 and Rht2 were aligned (atoms superimposed) to their corresponding closest sequence, the Rht1/CalG1 pair showed a 63 matching score and a 16.398 root mean square deviation (RMSD), while the Rht2/Alg13 pair had a 43 matching score and a 5.891 RMSD. We also selected the crystal structure of *Arabidopsis thaliana* rhamnosyltransferase UGT89C1 (PDB ID 6IJA; [59]). At the level of amino acid sequence, UGT89C1 does not share significant identity to neither Rht1 nor Rht2. Nevertheless, when its crystal structure was aligned to those of the predicted 3D models, the Rht1/UGT89C1 pair had a 58.5 matching score and a 7.799 RMSD while the Rht2/UGT89C1 pair had a 77 matching score and a 15.577 RMSD, both pairs having a better fit than when compared to their closest sequences (Figure 4C,D, respectively) (higher matching scores and lower RMSD are better). It is noteworthy that Rht2 has a global similarity to the structure of UGT89C1, while Rht1 has a good fit but only to one of the two Rossmann-like folds.

## 4. Discussion

The identification and classification of GTs is a complicated matter. These enzymes can be extremely specific on both the sugar donor and the acceptor molecule, but in many cases, the diversity in their sequences is not directly correlated to their specificity. Although there are only three known tridimensional folds for GTs (GT-A, GT-B, or GT-C) [55,56], these are multidomain proteins that show great diversity in their primary structures. Despite catalyzing closely related reactions, many of these transferases show little apparent sequence homology. The CAZY database classifies them into families (indicated as GTx) by amino acid sequence similarities, but the specificity of function and sequence homology for each family can be remarkably diverse [60,61]. The sequence diversity of these enzymes has been classified in at least 114 families (April 2022). Some families are monospecific for function (e.g., GT3 or GT19) and group just a small number of sequences with high amino acid similarities that include the totality of their catalytic domain. On the contrary, the shared amino acid homology in polyspecific families (e.g., GT1, GT2, or GT4) is limited to just isolated motifs of the catalytic domain, grouping a diverse and wide array of functions, such as cellulose and chitin synthase, glucosyltransferase, mannosyltransferase, rhamnosyltransferase, galactosyltransferase, and many others. Regardless of this variability in their primary structure, the three-dimensional fold of the enzymes within each family is expected to be of the same type (http://www.cazy.org/GlycosylTransferases.html, accessed on 25 April 2022): all members of family GT1 have a GT-B fold, those of family GT2 present a GT-A fold, and Glycosyltransferasases of family GT22 have a GT-C fold, to cite some examples [60,61]. It is worth mentioning that there are rhamnosyltransferases both in families GT1 (GT-B fold) and GT2 (GT-A fold) [60,61].

Their catalytic and recognition sites are the most conserved residues, but these are just short motifs shared with most members of the GT superfamily, such as the DxD motif found on those enzymes with a GT-A fold (NCBI CDD Conserved Protein Domain cl11394; [62]). This high similarity between very small fractions of their conserved residues allows for a very broad identification of an incomplete subset of the GT sequences present in a genome by use of traditional homology searches (e.g., BLAST). In addition to recovering just a subset, homology searches prevent the discrimination between the specific families. A more powerful and flexible bioinformatic tool to identify and discriminate GTs sequences is the use of Hidden Markov Model (HMM) profiles. Presently, many specific HMM profiles for GTs are available.

Accordingly, monosaccharide L-rhamnosyltransferases are multidomain proteins that show great diversity in their primary sequences. Their catalytic and recognition sites are the most conserved residues, but these are shared with most members of the glycosyltransferase superfamily (cl11394). For the particular case of rhamnosyltransferases, HMM profiles have been built, for the most part, only from prokaryotic sequences (pfam profile PF11316). Additionally, most of the prokaryotic rhamnosyltransferases have a GT-A fold (with a DxD motif present), while those from eukaryotes (viridiplantae) have a GT-B fold (which lack the DxD motif). The application of the prokaryotic HMM profiles to eukaryotic genomes often results in no results (false negatives) or misidentified genes (false positives). Currently, the standard for detecting L-rhamnosyltransferases by HMM are the TIGR01556 (Interpro, NCBI-TIGRFAMs) and pfam11316 (PFAM) probability matrices. No functional fungal rhamnosyltransferase gene has been described and biochemically tested, to date.

Currently, there are almost 61,500 putative rhamnosyltransferase protein sequences deposited on GenBank (although the number of unique sequences is closer to 14,000). For the gamma-proteobacteria class, there are reported about 11,500 L-rhamnosyltransferase sequences, of which 2581 are unique (NCBI, April 2022). These are distributed among multiple orders of gamma-proteobacteria. Being based on the gamma-proteobacteria class, the probability matrix hmm TIGR01556 is too specific, so its use on fungi (*S. schenckii* specifically) has turned out negative results. In eukaryotes, on the other hand, there are only about 400 sequences reported (mostly from plants), with only nine assigned to animals and nine to fungi. Only five of the fungal sequences are unique, with the other four belonging to different strains of the same genera. In all cases, the putative fungal rhamnosyltransferases were automatically annotated using the hmm profile PF11316. This profile was designed from a prokaryotic domain of unknown function, which belongs to a family of uncharacterized sequences (564 sequences) that includes a single sequence shown to be a rhamnosyltransferase from *Sphingomonas sp* ATCC 5319 [60,61]. Since GTs are multidomain proteins, many of these assignments of function are spurious. For example, one of the putative rhamnosyltransferases identified with this profile in *Neurospora crassa* (XP_958984.1) is also identified, with higher reliability, as a putative Heterokaryon incompatibility protein using the hmm profile PF06985. A similar situation occurs with the remaining four fungal sequences, making them all unlikely true fungal rhamnosyltransferases.

In addition to the PF11316 profile, there are available hmm profiles modeled from alignments of sequences recovered from alphaproteobacteria, gammaproteobacteria, and firmicutes, as well as a general profile modeled after the DxD motif. To overcome the high rate of false positives/false negatives limitation shown by the use of those profiles in fungi, we have designed a more specific ad hoc hmm profile based on eukaryotic (viridiplantae) rhamnosyltransferase sequences. As stated elsewhere, *S. schenckii* is known to secrete a heavily glycosylated glycoprotein, whose glycan portion is composed of *N*-linked mannose and rhamnose, but the rhamnosyltransferase gene required for its glycosylation has not been identified in its genome. Applying our hmm profile to its genome, we have identified five genes that were selected as encoding putative rhamnosyltransferases.

Molecular and biochemical tests were carried out to validate the predictions. Two out of the five candidate genes showed expression under lab growth conditions. These two genes were selected for further testing, cloning them for in vitro biochemical activity assays, confirming that both genes encoded for GTs with selectivity for rhamnose.

Finally, the protein sequences of the biochemically confirmed rhamnosyltransferases were subjected to an in silico structural analysis, showing that these proteins have a tridimensional structure belonging to those of glycosyltransferases of the GT-B fold type, but with differing characteristics. Rht2 has the typical two α/β/α Rossmann nucleotide binding domains, separated by a flexible linker and lacking the DxD motif that the majority of glycosyltransferases with a GT-A fold have in its active site that helps coordinate the metal ion and the nucleotide sugar. On the other hand, Rht1 appeared to have a single Rossmann-like fold, but its overall similarity is still closer to that of GT-B glycosyltransferases than to the GT-A fold ones. A couple of explanations may be drawn from these observations; one pointing to the possibility that it works as a dimer [63], or the other being that it presents a variant single-domain Rossmann-like fold, as previously reported for some GT-B glycosyltransferases [64]. These findings clearly separate the fungal rhamnosyltransferases from those found on prokaryotic cells. To the best of our knowledge, this is the first report of a functional rhamnosyltransferase, not only for *S. schenckii*, but for any fungal genome. Since rhamnose is not found on humans and other animals, this discovery widens the opportunities for the design of new antifungal drugs aimed at the biosynthesis of rhamnoconjugates [65].

## Figures and Tables

**Table 1:** Genes of putative rhamnosyltransferases identified on the *Sporothrix schenckii* genome.

GENE Symbol	mRNA AccnNumber	GeneID	Protein AccnNumber	Gene Description	Length
SPSK_01368	XM_016728283.1	gi|27663560	gb|XP_016590217.1	Sterol 3-beta-glucosyltransferase mRNA	1836
SPSK_04821	XM_016731584.1	gi|27666861	gb|XP_016583511.1	UDP-transferase mRNA	1415
SPSK_05538	XM_016732282.1	gi|27667559	gb|XP_016583713.1	beta-1,4-N-acetylglucosaminyltransferase mRNA	232
SPSK_05928	XM_016732636.1	gi|27667913	gb|XP_016591800.1	glucosylltransferase family 28 protein mRNA	920
SPSK_01110	XM_016728041.1	gi|27663318	gb|XP_016584143.1	hypothetical protein mRNA	576

**Table 2:** Domains detected for the putative rhamnosyltransferases identified on the *Sporothrix schenckii* genome.

Seq ID	aln Start	aln End	hmm Acc	hmm Name	Bitscore	Indiv. E-Value	Cond. E-Value	Clan
SPSK_05538	16	192	PF04101.19	Glyco_tran_28_C	57.35	2e-15	9.9e-20	CL0113
SPSK_05928	71	113	PF03033.23	Glyco_transf_28	32.35	9.2e-8	9.3e-12	CL0113
	479	549	PF00201.21	UDPGT	21.39	1e-4	1e-8	CL0113
SPSK_01368	1022	1128	PF02893.23	GRAM	71.46	5.6e-20	8.5e-24	CL0266
	1237	1372	PF03033.23	Glyco_transf_28	99.87	1.3e-28	2e-32	CL0113
	505	598	PF00169.32	PH	48.85	8.5e-13	1.3e-16	CL0266
SPSK_04821	415	576	PF03033.23	Glyco_transf_28	54.65	1.2e-14	1.2e-18	CL0113
	677	823	PF00201.21	UDPGT	30.15	2.3e-7	2.3e-11	CL0113
SPSK_01110	402	482	PF00201.21	UDPGT	26.23	3.5e-6	1.8e-10	CL0113

**Table 3:** Enzyme activity of recombinant Rht1 and Rht2 using different sugar donors.

	Empty Vector	rRth1	rRth2
No sugar donor	0.03 ± 0.02 *	0.01 ± 0.01	0.03 ± 0.02
+ UDP-Rhamnose	0.08 ± 0.06	198.7 ± 34.4 **	215.4 ± 28.6 **
+ UDP-Glucose	0.7 ± 0.4	0.9 ± 0.6	0.6 ± 0.4
+ GDP-Mannose	1.5 ± 0.7	2.1 ± 1.4	1.5 ± 0.8
+ UDP-N-Acetylglucosamine	0.3 ± 0.2	0.4 ± 0.2	0. 5 ± 0.3
+ UDP-Galactose	0.5 ± 0.3	0.9 ± 0.7	0.7 ± 0.4
+ UDP-Rhamnose and heat-inactivated protein	0.1 ± 0.09	0.1 ± 0.08	0.3 ± 0.2

* Expressed as trisaccharide min^−1^ mg protein^−1^. ** *p* < 0.05, when compared to the reaction with no sugar donor included.

## Data Availability

Not applicable.

## Supplementary Materials

The following supporting information can be downloaded at: https://www.mdpi.com/article/10.3390/jof8050529/s1, Supplementary Bioinformatics Methodologies: Bioinfo_Methods.docx; Figure S1: Confidence of predicted Alphafold2 3D models. Confidence values, expressed as pLDDT and color-coded as a traffic-light-like red-yellow-green gradient (with red being high, yellow average, and green low values) for Rht1 (A) and Rht2 (B). Confidence values, expressed as pLDDT and graphed per residue for Rht1 (C) and Rht2 (D); HMM profile 1: Rhamno_Transf_Euk.hmm.

## References

[1] Brown G.D., Denning D.W., Gow N.A., Levitz S.M., Netea M.G., White T.C. (2012). Hidden killers: Human fungal infections. Sci. Transl. Med..

[2] Fisher M.C., Gurr S.J., Cuomo C.A., Blehert D.S., Jin H., Stukenbrock E.H., Stajich J.E., Kahmann R., Boone C., Denning D.W. (2020). Threats posed by the fungal kingdom to humans, wildlife, and agriculture. mBio.

[3] Dalhoff A. (2018). Does the use of antifungal agents in agriculture and food foster polyene resistance development? A reason for concern. J. Glob. Antimicrob. Resist..

[4] Brauer V.S., Rezende C.P., Pessoni A.M., De Paula R.G., Rangappa K.S., Nayaka S.C., Gupta V.K., Almeida F. (2019). Antifungal agents in agriculture: Friends and foes of public health. Biomolecules.

[5] Du H., Bing J., Hu T., Ennis C.L., Nobile C.J., Huang G. (2020). *Candida auris*: Epidemiology, biology, antifungal resistance, and virulence. PLoS Pathog..

[6] Staniszewska M. (2020). Virulence Factors in *Candida* species. Curr. Protein Pept. Sci..

[7] Alspaugh J.A. (2015). Virulence mechanisms and *Cryptococcus neoformans* pathogenesis. Fungal Genet. Biol..

[8] Ardizzoni A., Wheeler R.T., Pericolini E. (2021). It takes two to tango: How a dysregulation of the innate immunity, coupled with *Candida* virulence, triggers VVC onset. Front. Microbiol..

[9] Tamez-Castrellón A.K., Romeo O., García-Carnero L.C., Lozoya-Pérez N.E., Mora-Montes H.M. (2020). Virulence factors in *Sporothrix schenckii*, one of the causative agents of sporotrichosis. Curr. Protein Pept. Sci..

[10] Lopes-Bezerra L.M., Walker L.A., Niño-Vega G., Mora-Montes H.M., Neves G.W.P., Villalobos-Duno H., Barreto L., Garcia K., Franco B., Martínez-Álvarez J.A. (2018). Cell walls of the dimorphic fungal pathogens *Sporothrix schenckii* and *Sporothrix brasiliensis* exhibit bilaminate structures and sloughing of extensive and intact layers. PLoS Negl. Trop. Dis..

[11] Díaz-Jiménez D.F., Pérez-García L.A., Martínez-Álvarez J.A., Mora-Montes H.M. (2012). Role of the fungal cell wall in pathogenesis and antifungal resistance. Curr. Fungal Infect. Rep..

[12] Klis F.M., de Groot P., Hellingwerf K. (2001). Molecular organization of the cell wall of *Candida albicans*. Med. Mycol..

[13] Latgé J.-P., Beauvais A., Chamilos G. (2017). The cell wall of the human fungal pathogen *Aspergillus fumigatus*: Biosynthesis, organization, immune response, and virulence. Ann. Rev. Microbiol..

[14] Martínez-Álvarez J.A., Pérez-García L.A., Mellado-Mojica E., López M.G., Martínez-Duncker I., Lópes-Bezerra L.M., Mora-Montes H.M. (2017). *Sporothrix schenckii sensu stricto* and *Sporothrix brasiliensis* are differentially recognized by human peripheral blood mononuclear cells. Front. Microbiol..

[15] Lozoya-Pérez N.E., Clavijo-Giraldo D.M., Martínez-Duncker I., García-Carnero L.C., López-Ramírez L.A., Niño-Vega G.A., Mora-Montes H.M. (2020). Influences of the culturing media in the virulence and cell wall of *Sporothrix schenckii*, *Sporothrix brasiliensis*, and *Sporothrix globosa*. J. Fungi.

[16] Hoyer L.L., Cota E. (2016). *Candida albicans* Agglutinin-Like Sequence (Als) family vignettes: A review of Als protein structure and function. Front. Microbiol..

[17] Staniszewska M., Bondaryk M., Ochal Z. (2017). Contribution of aspartic proteases in *Candida* virulence. protease inhibitors against candida infections. Curr. Protein Pept. Sci..

[18] Lopes-Bezerra L.M. (2011). *Sporothrix schenckii* cell wall peptidorhamnomannans. Front. Microbiol..

[19] García-Carnero L.C., Salinas-Marín R., Lozoya-Pérez N.E., Wrobel K., Wrobel K., Martínez-Duncker I., Niño-Vega G.A., Mora-Montes H.M. (2021). The heat shock protein 60 and Pap1 participate in the *Sporothrix schenckii*-host interaction. J. Fungi.

[20] Mora-Montes H.M., Ponce-Noyola P., Villagómez-Castro J.C., Gow N.A.R., Flores-Carreón A., López-Romero E. (2009). Protein glycosylation in *Candida*. Future Microbiol..

[21] Lozoya-Pérez N.E., Casas-Flores S., de Almeida J.R.F., Martínez-Álvarez J.A., López-Ramírez L.A., Jannuzzi G.P., Trujillo-Esquivel E., Estrada-Mata E., Almeida S.R., Franco B. (2019). Silencing of *OCH1* unveils the role of *Sporothrix schenckii N*-linked glycans during the host-fungus interaction. Infect. Drug Resist..

[22] Perez-Nadales E., Nogueira M.F., Baldin C., Castanheira S., El Ghalid M., Grund E., Lengeler K., Marchegiani E., Mehrotra P.V., Moretti M. (2014). Fungal model systems and the elucidation of pathogenicity determinants. Fungal Genet. Biol..

[23] Gómez-Gaviria M., Vargas-Macías A.P., García-Carnero L.C., Martínez-Duncker I., Mora-Montes H.M. (2021). Role of protein glycosylation in interactions of medically relevant fungi with the host. J. Fungi.

[24] Santhanam P., Boshoven J.C., Salas O., Bowler K., Islam M.T., Saber M.K., van den Berg G.C., Bar-Peled M., Thomma B.P. (2017). Rhamnose synthase activity is required for pathogenicity of the vascular wilt fungus *Verticillium dahliae*. Mol. Plant Pathol..

[25] Lopes L.C., da Silva M.I., Bittencourt V.C., Figueiredo R.T., Rollin-Pinheiro R., Sassaki G.L., Bozza M.T., Gorin P.A., Barreto-Bergter E. (2011). Glycoconjugates and polysaccharides from the *Scedosporium*/*Pseudallescheria boydii* complex: Structural characterisation, involvement in cell differentiation, cell recognition and virulence. Mycoses.

[26] Tamez-Castrellón A.K., van der Beek S.L., López-Ramírez L.A., Martínez-Duncker I., Lozoya-Pérez N.E., van Sorge N.M., Mora-Montes H.M. (2021). Disruption of protein rhamnosylation affects the *Sporothrix schenckii*-host interaction. Cell Surf..

[27] Villalobos-Duno H.L., Barreto L.A., Alvarez-Aular Á., Mora-Montes H.M., Lozoya-Pérez N.E., Franco B., Lopes-Bezerra L.M., Niño-Vega G.A. (2021). Comparison of cell wall polysaccharide composition and structure between strains of *Sporothrix schenckii* and *Sporothrix brasiliensis*. Front. Microbiol..

[28] Lopes-Bezerra L.M., Mora-Montes H.M., Zhang Y., Nino-Vega G., Rodrigues A.M., de Camargo Z.P., de Hoog S. (2018). Sporotrichosis between 1898 and 2017: The evolution of knowledge on a changeable disease and on emerging etiological agents. Med. Mycol..

[29] Mora-Montes H.M., Dantas Ada S., Trujillo-Esquivel E., de Souza Baptista A.R., Lopes-Bezerra L.M. (2015). Current progress in the biology of members of the *Sporothrix schenckii* complex following the genomic era. FEMS Yeast Res..

[30] Teixeira M.M., de Almeida L.G., Kubitschek-Barreira P., Alves F.L., Kioshima E.S., Abadio A.K., Fernandes L., Derengowski L.S., Ferreira K.S., Souza R.C. (2014). Comparative genomics of the major fungal agents of human and animal Sporotrichosis: *Sporothrix schenckii* and *Sporothrix brasiliensis*. BMC Genom..

[31] Bohl T., Bai L., Li H. (2021). Recent progress in structural studies on the GT-C superfamily of protein glycosyltransferases. Sub-Cell. Biochem..

[32] Breton C., Fournel-Gigleux S., Palcic M.M. (2012). Recent structures, evolution and mechanisms of glycosyltransferases. Curr. Opin. Struct. Biol..

[33] Breton C., Snajdrová L., Jeanneau C., Koca J., Imberty A. (2006). Structures and mechanisms of glycosyltransferases. Glycobiology.

[34] Giosa D., Felice M.R., Giuffrè L., Aiese Cigliano R., Paytuví-Gallart A., Lo Passo C., Barresi C., D’Alessandro E., Huang H., Criseo G. (2020). Transcriptome-wide expression profiling of *Sporothrix schenckii* yeast and mycelial forms and the establishment of the Sporothrix Genome DataBase. Microb. Genom..

[35] Sayers E.W., Bolton E.E., Brister J.R., Canese K., Chan J., Comeau D.C., Connor R., Funk K., Kelly C., Kim S. (2022). Database resources of the National Center for Biotechnology Information. Nucleic Acids Res..

[36] Mistry J., Chuguransky S., Williams L., Qureshi M., Salazar G.A., Sonnhammer E.L.L., Tosatto S.C.E., Paladin L., Raj S., Richardson L.J. (2021). Pfam: The protein families database in 2021. Nucleic Acids Res..

[37] Berman H.M., Westbrook J., Feng Z., Gilliland G., Bhat T.N., Weissig H., Shindyalov I.N., Bourne P.E. (2000). The Protein Data Bank. Nucleic Acids Res..

[38] Burley S.K., Bhikadiya C., Bi C., Bittrich S., Chen L., Crichlow G.V., Christie C.H., Dalenberg K., Di Costanzo L., Duarte J.M. (2021). RCSB Protein Data Bank: Powerful new tools for exploring 3D structures of biological macromolecules for basic and applied research and education in fundamental biology, biomedicine, biotechnology, bioengineering and energy sciences. Nucleic Acids Res..

[39] Jumper J., Evans R., Pritzel A., Green T., Figurnov M., Ronneberger O., Tunyasuvunakool K., Bates R., Dek A., Potapenko A. (2021). Highly accurate protein structure prediction with AlphaFold. Nature.

[40] Mitchell A.L., Almeida A., Beracochea M., Boland M., Burgin J., Cochrane G., Crusoe M.R., Kale V., Potter S.C., Richardson L.J. (2019). MGnify: The microbiome analysis resource in 2020. Nucleic Acids Res..

[41] Mirdita M., von den Driesch L., Galiez C., Martin M.J., Sding J., Steinegger M. (2017). Uniclust databases of clustered and deeply annotated protein sequences and alignments. Nucleic Acids Res..

[42] Mirdita M., Steinegger M., Sding J. (2019). MMseqs2 desktop and local web server app for fast, interactive sequence searches. Bioinformatics.

[43] Mirdita M., Schütze K., Moriwaki Y., Heo L., Ovchinnikov S., Steinegger M. (2022). ColabFold-Making protein folding accessible to all. bioRxiv.

[44] Katoh K., Rozewicki J., Yamada K.D. (2019). MAFFT online service: Multiple sequence alignment, interactive sequence choice and visualization. Brief. Bioinform..

[45] Trujillo-Esquivel E., Martínez-Álvarez J.A., Clavijo-Giraldo D.M., Hernández N.V., Flores-Martínez A., Ponce-Noyola P., Mora-Montes H.M. (2017). The *Sporothrix schenckii* gene encoding for the ribosomal protein L6 has constitutive and stable expression and works as an endogenous control in gene expression analysis. Front. Microbiol..

[46] Robledo-Ortiz C.I., Flores-Carreón A., Hernández-Cervantes A., Álvarez-Vargas A., Lee K.K., Díaz-Jiménez D.F., Munro C.A., Cano-Canchola C., Mora-Montes H.M. (2012). Isolation and functional characterization of *Sporothrix schenckii ROT2*, the encoding gene for the endoplasmic reticulum glucosidase II. Fungal Biol..

[47] Trujillo-Esquivel E., Franco B., Flores-Martínez A., Ponce-Noyola P., Mora-Montes H.M. (2016). Purification of single-stranded cDNA based on RNA degradation treatment and adsorption chromatography. Nucleosides Nucleotides Nucleic Acids.

[48] Livak K.J., Schmittgen T.D. (2001). Analysis of relative gene expression data using real-time quantitative PCR and the 2(-Delta Delta C(T)) Method. Methods.

[49] Martínez-Álvarez J.A., García-Carnero L.C., Kubitschek-Barreira P.H., Lozoya-Pérez N.E., Belmonte-Vázquez J.L., de Almeida J.R., Gómez-Infante A.d.J., Curty N., Villagómez-Castro J.C., Peña-Cabrera E. (2019). Analysis of some immunogenic properties of the recombinant *Sporothrix schenckii* Gp70 expressed in *Escherichia coli*. Future Microbiol..

[50] Chevallet M., Luche S., Rabilloud T. (2006). Silver staining of proteins in polyacrylamide gels. Nat. Protoc..

[51] Yonekura-Sakakibara K., Tohge T., Niida R., Saito K. (2007). Identification of a flavonol 7-O-rhamnosyltransferase gene determining flavonoid pattern in *Arabidopsis* by transcriptome coexpression analysis and reverse genetics. J. Biol. Chem..

[52] Mora-Montes H.M., López-Romero E., Zinker S., Ponce-Noyola P., Flores-Carreón A. (2004). Hydrolysis of Man_9_GlcNAc_2_ and Man_8_GlcNAc_2_ oligosaccharides by a purified α-mannosidase from *Candida albicans*. Glycobiology.

[53] Plaine A., Walker L., Da Costa G., Mora-Montes H.M., McKinnon A., Gow N.A., Gaillardin C., Munro C.A., Richard M.L. (2008). Functional analysis of *Candida albicans* GPI-anchored proteins: Roles in cell wall integrity and caspofungin sensitivity. Fungal Genet. Biol..

[54] Hernández N.V., López-Ramírez L.A., Díaz-Jiménez D.F., Mellado-Mojica E., Martínez-Duncker I., López M.G., Mora-Montes H.M. (2017). *Saccharomyces cerevisiae KTR4*, *KTR5* and *KTR7* encode mannosyltransferases differentially involved in the *N*- and *O*-linked glycosylation pathways. Res. Microbiol..

[55] Coutinho P.M., Deleury E., Davies G.J., Henrissat B. (2003). An evolving hierarchical family classification for glycosyltransferases. J. Mol. Biol..

[56] Gloster T.M. (2014). Advances in understanding glycosyltransferases from a structural perspective. Curr. Opin. Struct. Biol..

[57] Chang A., Singh S., Helmich K.E., Goff R.D., Bingman C.A., Thorson J.S., Phillips G.N. (2011). Complete set of glycosyltransferase structures in the calicheamicin biosynthetic pathway reveals the origin of regiospecificity. Proc. Natl. Acad. Sci. USA.

[58] Wang X., Weldeghiorghis T., Zhang G., Imperiali B., Prestegard J.H. (2008). Solution structure of Alg13: The sugar donor subunit of a yeast N-acetylglucosamine transferase. Structure.

[59] Zong G., Fei S., Liu X., Li J., Gao Y., Yang X., Wang X., Shen Y. (2019). Crystal structures of rhamnosyltransferase UGT89C1 from *Arabidopsis thaliana* reveal the molecular basis of sugar donor specificity for UDP-β-l-rhamnose and rhamnosylation mechanism. Plant J. Cell Mol. Biol..

[60] Campbell J.A., Davies G.J., Bulone V., Henrissat B. (1997). A classification of nucleotide-diphospho-sugar glycosyltransferases based on amino acid sequence similarities. Biochem. J..

[61] Drula E., Garron M.L., Dogan S., Lombard V., Henrissat B., Terrapon N. (2022). The carbohydrate-active enzyme database: Functions and literature. Nucleic Acids Res..

[62] Wiggins C.A., Munro S. (1998). Activity of the yeast MNN1 α-1,3-mannosyltransferase requires a motif conserved in many other families of glycosyltransferases. Proc. Natl. Acad. Sci. USA.

[63] Kellokumpu S., Hassinen A., Glumoff T. (2016). Glycosyltransferase complexes in eukaryotes: Long-known, prevalent but still unrecognized. Cell. Mol. Life Sci. CMLS.

[64] Grewal R.K., Shaikh A.R., Gorle S., Kaur M., Videira P.A., Cavallo L., Chawla M. (2021). Structural insights in mammalian sialyltransferases and fucosyltransferases: We have come a long way, but it is still a long way down. Molecules.

[65] Wagstaff B.A., Zorzoli A., Dorfmueller H.C. (2021). NDP-rhamnose biosynthesis and rhamnosyltransferases: Building diverse glycoconjugates in nature. Biochem. J..

