# Supplementary material for: The Search for Cryptic L-Rhamnosyltransferases on the Sporothrix schenckii Genome
Source: J Fungi (Basel). 2022 May 20;8(5):529. doi: 10.3390/jof8050529 (PMC9145935; doi:10.3390/jof8050529)
Supplement: Supplementary file 1 [file jof-08-00529-s001.zip › Supplementary Materials Bioinformatics Methodologies.pdf]

Supplementary Bioinformatics Methodologies: Bioinfo_Methods.docx

2.1. Hardware and software environment used

All locally run bioinformatics analysis were conducted on an HP workstation with 8 Intel® Core™ i7-6700 CPU @ 3.40GHz, 16 Gb of RAM, 1 TB SSD, and the Ubuntu 20.04.4 LTS operating system. Where required, analyses were run on publicly accessible servers: NCBI databases [35], PFAM database [36], RCSB PDB database and mmseqs2 tool [37,38], and AlphaFold2 [39] mounted on two Google Colab projects: AlphaFold2.ipynb and AlphaFold.ipynb [39-43].

2.2. Sporothrix schenckii proteome and PFAM/TIGRFAM Hidden Markov Models profiles

The 10,293 proteins of the sequenced *S. schenckii* (ATCC MYA-4821) genome [30] were downloaded from NCBI database as a FASTA file (Bioproject PRJNA218070) and named `Ssprot.faa`.

Available HMM profiles for rhamnosyltransferases were downloaded from PFAM and TIGRFAM: PF11316 Rhamno_transf (alphaproteobacteria) and TIGR01556 rhamnosyltran (gammaproteobacteria). Also, available HMM profiles for general glycosyltransferases were downloaded from PFAM and TIGRFAM: PF04488 (DxD motif) and TIGR01426 (glycosyltrasnferase, MGT family).

2.3. Construction of ad hoc eukaryotic Rhamnosyltransferases HMM profile and hmmer searches

37 protein sequences of reported eukaryotic rhamnosyltransferases from viridiplantae were downloaded from NCBI database and saved as Viri_rhamn.fas. A Multiple Sequence Alignment (MSA) was constructed with the locally installed command line MAFFT algorithm (v.7.450) [44] .

```
    mafft --ep 0 --genafpair --maxiterate 1000 --
thread -1 Viri_rhamn.fas > vr37.fasta
```

Twenty-one sequences were discarded due to being partial, identical to other sequences, or highly divergent. The remaining sixteen sequences were realigned with the MAFFT algorithm (same parameters as above) and the resulting MSA saved in aligned FASTA format (`vr16.fasta`).

Accession numbers: NP_564357.1, ACX70154.1, BAC43110.1, AEE31240.1, AAL06646.2, AFB73772.1, Q8GVE3.2, XP_004485549.1, XP_004485550.1, NP_001274799.1, Q9S9P6.1, CAB78073.1, Q66PF2.1, ABB84472.1, BAA98174.1, and XP_014517591.1.

An *ad hoc* HMM profile was generated from the MSA of the sixteen eukaryotic rhamnosyltransferases with the *hmmbuild* command of locally installed HMMER (v.3.3) command line tool.

```
    hmmbuild Rhamno_Transf_Euk.hmm vr16.fasta
```

Searches with PFAM/TIGRFAM HMM profiles as well as the *ad hoc* HMM profile were conducted on the *S. schenckii* proteome using the *hmmsearch* command of the locally installed HMMER (v.3.3) command line tool. The *S. schenckii* proteome in FASTA format (*Ssprot.faa*) was used as the target database.

```
hmmsearch -o Ss_rhamno_output.txt HMMprofile.hmm Ssprot.faa
```

Same parameters were used for all searches only changing the HMM profile used and the corresponding output file name.

To determine putative domains present on the recovered sequences, these were submitted as a multiple sequence FASTA file to the Batch sequence search tool at the PFAM database, with an E-value cut-off of 1.0.

2.4. In silico structural analysis of putative rhamnosyltransferases proteins

Retrieval of closest crystalized proteins of Rht1 and Rht2 from the RCSB PDB database was done using its advanced sequence search tool. Default parameters were used except for the "Return" value that was changed to "Polymer Entities".

3D modeling of Rht1 and Rht2 proteins was carried out at publicly available implementations of the Alphafold2 algorithm [39] mounted on the Google Colab services. We used both the AlphaFold2.ipynb (ColabFold: AlphaFold2 using MMseqs2) as well as the AlphaFold.ipynb (AlphaFold Colab). These implementations differ on the method for multiple sequence alignment used: MMseqs2 and jackhammer, respectively. All defaults parameters were used, and no templates were selected. Best models were selected according to a combination of the following result values: number of sequences per position, predicted IDDT per position (pLDDT), and predicted Alignment Error (pAE).

A locally installed implementation of Pymol (v.3.2) was used to visualize the 3D protein models uploaded as pdb files. Secondary structures were colored using the default color-scheme (alpha helixes red, beta sheets yellow, coils green). Confidence values for each atom, expressed as pLDDT values from 0 to 100 and included on pdb files, were shown using the default color-scheme (traffic-light-like red-yellow-green gradient).

Alignments of 3D models and crystalized proteins (atoms superimposition) were conducted also on Pymol with the following command:

align structure1, structure 2

where structure1 and structure2 were changed accordingly to the corresponding molecule file name. Matching scores and Root Mean Square Deviation (RMSD) values were recorded for each pair of structures.
