# Supplementary material for: The Search for Cryptic L-Rhamnosyltransferases on the Sporothrix schenckii Genome
Source: J Fungi (Basel). 2022 May 20;8(5):529. doi: 10.3390/jof8050529 (PMC9145935; doi:10.3390/jof8050529)
Supplement: Supplementary file 1 [file jof-08-00529-s001.zip › Supplementary Materials Figure S1.pdf]

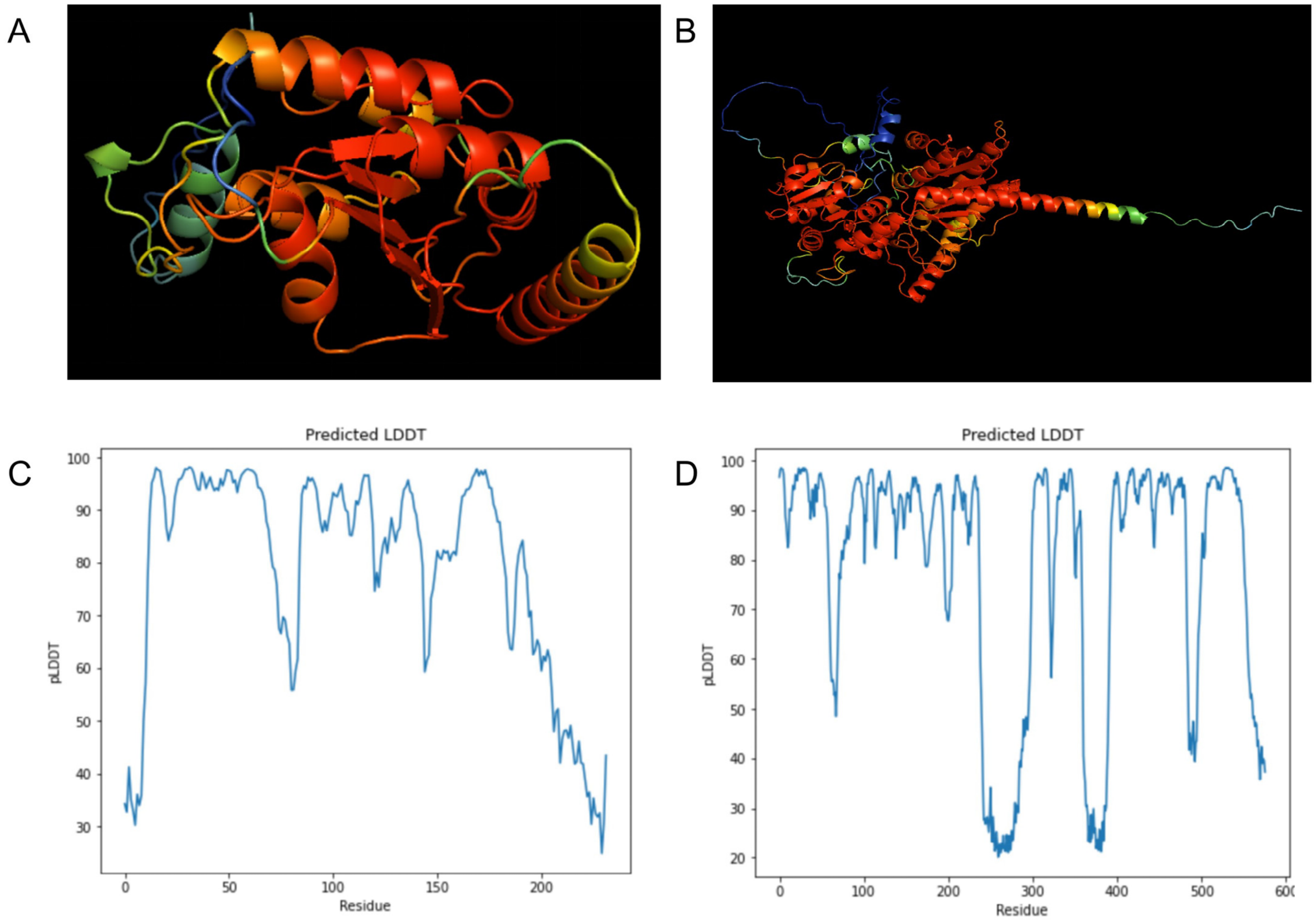

Figure S1. Confidence of predicted Alphafold2 3D models. Confidence values, expressed as pLDDT and color-coded as a traffic-light-like red-yellow-green gradient (with red being high, yellow average, and green low values) for Rht1 (**A**) and Rht2 (**B**). Confidence values, expressed as pLDDT and graphed per residue for Rht1 (**C**) and Rht2 (**D**).
